# Volatile profiling as a potential biochemical marker for validation of gamma irradiation derived putative mutants in polyembryonic genotypes of mango (*Mangifera indica* L.)

**DOI:** 10.3389/fpls.2023.1168947

**Published:** 2023-09-01

**Authors:** Nusrat Perveen, M. R. Dinesh, M. Sankaran, K. S. Shivashankara, K. V. Ravishankar, R. Venugopal, Hidayatullah Mir

**Affiliations:** ^1^ Division of Fruit Crops, Indian Council of Agricultural Research (ICAR)-Indian Institute of Horticultural Research, Bengaluru, India; ^2^ Division of Basic Sciences, Indian Council of Agricultural Research (ICAR)-Indian Institute of Horticultural Research, Bengaluru, India; ^3^ Division of Social Sciences and Training, Indian Council of Agricultural Research (ICAR)-Indian Institute of Horticultural Research, Bengaluru, India; ^4^ Department of Horticulture (Fruit and Fruit Technology), Bihar Agricultural University, Bhagalpur, India

**Keywords:** mango, polyembryony, mutation, nucellar seedlings, SSR markers, volatile profiling

## Abstract

**Introduction:**

Putative mutants were generated through gamma irradiation in the polyembryonic mango genotype Nekkare. The putative mutant progenies along with control seedlings and mother plants were evaluated by comparing the compositions and relative proportions of their major volatile compounds.

**Methods:**

Volatile profiling was done using headspace-solid phase micro-extraction (HS SPME) method coupled with gas chromatography–mass spectrometry (GC-MS MS). Furthermore, characterisation of putative mutants and control seedlings was carried out using simple sequence repeat (SSR) markers to ascertain the genetic diversity present in the samples under study.

**Results:**

Monoterpenes were the most abundant volatile compound in all the studied samples (ranging from 34.76% to 91.41%) out of which I-Phellandrene and cis-Ocimene formed the major fraction in mother plants (20.45%–21.86% and 16.17%–21.27%, respectively) and control seedlings (23.32%–24.95% and 18.95%–20.81%, respectively), while beta-Phellandrene was dominant in the selected putative mutant samples (2.34%–29.53%). Among sesquiterpenes, trans-Caryophyllene was detected only in the putative mutant samples (0.10%–30.18%). Grouping together of mother plants and control seedlings was seen in the cluster analysis, while the putative mutants grouped apart from them suggesting genetic diversity. Genetic distance between the mother plants and control seedlings ranged from 0.97 to 2.73, while between putative mutants, control seedlings, and mother plants, it ranged from 6.54 to 9.82. SSR-based characterisation of putative mutant seedlings showed that mutation caused variability in the treated population. This was evident from the high allelic richness ranging from 4 to 12 with a mean of 7 and a higher mean Shannon’s Information Index (1.50) of the putative mutant population.

**Discussion:**

The study demonstrates that volatile profiling and molecular characterisation using SSR markers could be used as a tool to detect variation in a mutated population. In addition, volatile profiling can be used to validate putative mutants in polyembryonic mango genotypes where the seedlings of nucellar origin are similar to mother plants.

## 1 Introduction

Polyembryony is a trait in certain mango genotypes where multiple apomictic embryos develop from the maternal nucellar tissues along with a single zygotic embryo (Asker and Jerling, 1992). The plants originating from apomictic embryos, also known as nucellar seedlings, are “true-to-type.” The polyembryonic genotypes are ideal as rootstocks, as they are uniform owing to their “true-to-type” nature. However, the use of these genotypes in breeding programme is often limited by their narrow genetic base. Hence, creating variability in these genotypes for various desirable traits, *viz.*, plant stature and salinity tolerance, would make them highly suitable as rootstocks. Induced mutations have been widely employed for creating variability in heterozygous crops like mango (Rossetto et al., 1997; Karsinah et al., 2012; Kumar et al., 2018; Rime et al., 2019).

In polyembryonic mango genotypes, the vigorous and first emerging seedlings, which grow well until maturity, are reported to be of nucellar origin (Srivastava et al., 1988). These nucellar seedlings would be similar to mother plants. The variation observed in the nucellar seedlings upon gamma irradiation can be considered to be due to mutation. Several studies in the past were used to validate mutants including molecular markers like microsatellite markers (Damasco et al., 2019), inter-simple sequence repeats (ISSR) markers (Due et al., 2019), phenotypic characterisation (Atay et al., 2019), and stomatal characters (Yasmeen et al., 2020). Volatile profiling was used as a biochemical marker for the characterisation of germplasm in fig (Oliveira et al., 2010), mango (Li et al., 2017), avocado (Ali et al., 2020), and apple (Roberts and Spadafora, 2020) owing to its species, cultivar specificity, and stability under different environmental conditions. Volatile compounds have also been used as biomarkers for determining susceptibility of different *Mangifera indica* genotypes to mango gall fly (Augustyn et al., 2010) and for distinguishing between the fruit type and the pickling type of mango (Vasugi et al., 2012).

In our previous study, a high level of variability was observed for both morphological and stomatal traits in the putative mutant population of Nekkare (Perveen et al., 2022). Hence, in this study, genetic diversity analysis was carried out in putative mutants of Nekkare exhibiting maximum morphological variability using simple sequence repeat (SSR) markers. Gamma irradiation is likely to cause mutation through deletion and is more likely to induce small deletions between 1 and 10 kbp (Morita et al., 2009). Hence, DNA markers can effectively be used to detect these variations. In this study, putative mutant seedlings generated by gamma irradiation were validated by comparing the leaf volatile profiles of mother plants, control seedlings, and selected putative mutants of polyembryonic mango genotype Nekkare. Our hypotheses were as follows: i) being nucellar in nature, the control seedlings will be similar to mother plants, and any variation observed in the seedlings emerging from gamma irradiation treated kernels could be considered to have been a result of mutation, and ii) SSR markers can be useful in determining whether the morphological diversity in the putative mutant population has manifested at genetic level or not.

## 2 Materials and methods

### 2.1 Experimental material

This study was a part of mango rootstock breeding programme undergoing at ICAR-Indian Institute of Horticultural Research (IIHR), Bengaluru, India. For gamma irradiation, fruits were collected from 30 years old Nekkare mother plants (NMP) being maintained in the Field Gene Bank of IIHR. The putative mutant population was generated by irradiating the seed kernels extracted from these fruits with five doses of gamma irradiation, *viz.*, T_1_: 15 Gy, T_2_: 20 Gy, T_3_: 25 Gy, T_4_: 30 Gy, and T_5_: 35 Gy. Polyembryonic genotypes produce more than one seedling per seed. The putative mutant seedlings obtained after sowing the irradiated seed kernels were first evaluated for determining the radio-sensitivity and LD_50_ dose (Perveen et al., 2023a). Higher doses of gamma irradiation resulted in decreased number in seedlings per seed, and only the vigorous seedlings were found to survive (Perveen et al., 2023a). Two months after germination, multiple seedlings emerging from each seed kernel were separated and transplanted in new polybags.

Six months after germination, only one to two seedlings survived per seed, which were considered to be nucellar in origin due to the vigour taking cognisance of the previous study (Srivastava et al., 1988). At this stage, a total of 100 randomly selected putative mutants (20 from each treatment) and 20 control seedlings were screened for morphological and stomatal parameters. On the basis of coefficient of variability (CV), T_5_: 35 Gy irradiation treatment was found to result in maximum morphological diversity (Perveen et al., 2022). Hence, the putative mutants generated through 35 Gy gamma irradiation were used for the present study. Leaf volatile profiling was carried out for two Nekkare mother plants (NMP_1_ and NMP_2_) and 9 months old, 18 control seedlings (NC) along with 18 putative mutants (NM).

### 2.2 HS-SPME extraction of volatiles

Recently matured leaves from NMP, NC, and NM were collected, wrapped separately in aluminium foil after proper labelling, and flash frozen in liquid nitrogen. Owing to the limitation of resources and to reduce the cost of volatile profiling, the samples for analysis were made by mixing leaves from more than one seedling. A total of 18 control seedlings were divided into three samples, *viz.*, NC_1_, NC_2_, and NC_3,_ each sample consisting of leaves from six seedlings. A total of 18 putative mutants were divided into six samples, each consisting of leaves from three putative mutant seedlings. These samples were as follows: NM_1_, consisting seedlings of 10–15 cm (dwarf); NM_2_, having seedlings of 16–25 cm (moderately vigorous); NM_3_, comprising seedlings of >26 cm height (vigorous); NM_4_, comprised of seedlings with narrow leaves (leaf blade width <3 cm); NM_5_, comprised of seedlings with broad leaves (leaf blade width ≥3 cm); and NM_6_, comprised of seedlings with dark green leaf.

The volatile compounds were analysed using headspace-solid phase micro-extraction (HS-SPME) method, which involved submerging a thin fused-silica fibre covered with the extracting phase in the headspace of the samples being analysed in order to extract the analytes onto it. It is made of a fused silica fibre that is 1–2 cm long and coated with a stationary phase, such as polydimethylsiloxane (PDMS), divinyl benzene (DVB), carboxen (CAR), or a mixture of all the above three compounds, bonded to a stainless-steel plunger and holder. These fibres are first conditioned at 250°C for 2–3 h in the injector port of GC with a continued flow of helium gas. For extraction of volatiles, 5 g of properly ground leaf sample was taken in a 150-ml conical flask containing a magnetic stirrer, and the mouth of conical flask was properly covered with silicon stopper. This step was followed by insertion of a previously conditioned SPME fibre in the conical flask to adsorb the headspace volatiles for 2 h.

### 2.3 Gas chromatography and mass spectrometry

The SPME fibre was pumped into the gas chromatography–mass spectrometry (GC-MS) injector port after 2 h, and the adsorbed volatiles were allowed to desorb for 10 min. A Varian-3800 gas chromatograph and a Varian-4000 mass spectrometer with an ion trap mass selective detector were used to conduct the GC-MS analysis. The fused-silica capillary column used for the analysis had the following dimensions: 30mm × 0.25mm, 0.25mm film thickness. The column temperature programmes were 40°C for 2 min at an increment of 3°C/min to 190°C with a hold for 1 min, followed by an increment of 5°C/min to 220°C. The injector temperature was set at 250°C, and all injections were made in split-less mode for 0.2 min.

The spectrometer was operating in external electron ionisation mode, with helium serving as the carrier gas (1.5ml/min), the injector temperature being 250°C, the trap temperature being 180°C, the ion source heating being 190°C, the transfer line heating being 260°C, the EI mode being at 70eV, and the full scan range being 50–350amu. The total chromatogram for each sample was obtained by adding all the GC peak areas, and each volatile compound was expressed as relative per cent area. By comparing the collected mass spectra to the spectra found in the Wiley and NIST-2007 libraries, the volatile compounds were identified by their retention periods with reference to standard compounds. The analysis of all the samples was done in triplicate. The results correspond to mean of three readings, generally followed for SPME analysis (Fernandes et al., 2009).

### 2.4 Molecular characterisation of selected putative mutants

Young healthy leaves were taken from 9-month-old, 20 putative mutants (generated through 35Gy gamma irradiation) and 20 untreated (control) seedlings. The collected leaves were wiped with 70% (v/v) alcohol and stored in butter paper envelopes at −10°C until DNA isolation. DNA isolation was done using modified Cetyltrimethylammonium bromide (CTAB) method (Ravishankar et al., 2000). Using Nanodrop, the absorbance at 260 and 280nm was measured to assess the DNA content, and the ratio of the absorbance at 260–280nm (A_260_/A_280_) was used to quantify the purity of the DNA. The extracted genomic DNA samples were analysed on a 0.8% (w/v) agarose gel using 0.5× Tris-acetate-EDTA (TAE) buffer to perform a qualitative examination of the DNA.

Fluorescence-based PCR method was used for fast, efficient, and precise amplification of microsatellites (Schuelke, 2000), carried out in 20**-**µl reaction volume. Previously, 90 genomic SSR markers were developed using next-generation sequencing technology (Ravishankar et al., 2015). These markers exhibited high polymorphism information content (PIC) values ranging from 0.738 to 0.960 with a mean of 0.899 and showed high transferability within the *Mangifera* genus (Ravishankar et al., 2015). From these 90 markers, 12 most informative SSR markers as shown by subsequent studies (data not given) were used for this study. The details of SSR primers, components of PCR reaction mixture along with their concentration, and the PCR protocol followed are presented in **Supplementary Tables S1.1, S1.2**, and **S1.3**, respectively. The amplification of PCR products was first confirmed by separating them on 3% (w/v) agarose gel. Once the amplification of PCR products was confirmed, four PCR products labelled with four different fluorophores, *viz.*, FAM, VIC, NED, and PET, were mixed to get a single sample. Genotyping of these samples was then performed using an automatic 96-capillary automated DNA sequencer (ABI 3730 DNA Analyser, Applied Biosystems, USA) at ICRISAT facility, Hyderabad, India.

### 2.5 Statistical analysis

#### 2.5.1 Volatile profiling

After applying Squared Euclidian Cluster analysis to all of the characters’ means, Ward’s approach was used to create a dendrogram (Rencher, 1995). From the Euclidean distances, a Pearson correlation matrix was created using PROC CLUSTER and PROC PRINCOMP of SAS V 9.3. Principal components analysis (PCA) was then performed on the correlation matrix (SAS, 2012). A two-dimensional graph was used to plot the first two main principal components.

#### 2.5.2 Analysis of molecular data and genetic diversity

Samples were separated on the automatic capillary automated DNA Sequencer once the PCR product’s amplification was validated. Peak Scanner V1.0 software (Applied Biosystems) was used to analyse and compile the raw data to determine the allele size (in base pairs). The obtained results were further employed for genetic analysis using CERVUS 3.0.3 software (Kalinowski et al., 2007). Using GenAIEx 6.5, genetic diversity metrics for each group were calculated, including allelic richness, heterozygosity, Shannon’s Information Index, and fixation index (Peakall and Smouse, 2006; Peakall and Smouse, 2012). The Gower similarity coefficient served as the input for a dendrogram/phylogenetic tree constructed using the unweighted pair-group method with arithmetic mean (UPGMA) clustering option of the PAST 4.0 software (Hammer et al., 2001).

## 3 Results

### 3.1 Volatile profiling

The compositions and proportions of major volatile compounds (VCs) present in the leaf samples of mother plants, control, and putative mutants determined by GC-MS-MS analysis is presented in **Table 1**. In this study, monoterpenes and sesquiterpenes were found to be the main VCs present in all the studied samples. α-Pinene was detected in all the selected putative mutant samples except in NM_2_. Camphene was detected in two putative mutant samples, NM_2_ (15.82%) and NM_3_ (22.52%), and trans-Ocimene was detected in NM_5_ (0.3%) and NM_6_ (0.84%), while these were absent in all other samples under study. Sabinene, which was present in all the mother plants and control seedlings, was detected only in three of the six putative mutant samples, NM_4_ (0.41%), NM_5_ (0.54%), and NM_6_ (0.88%). Similarly, beta-Ocimene, 3-Carene, γ-Terpinene, and α-Terpinolene were present only in a few putative mutant samples, while they were not detected in either mother plants or control samples. Furthermore, I-Phellandrene was one of the major monoterpenes (more than 20%) detected in all the mother plants and control samples and was also present in higher amount in two of the selected putative mutant samples, NM_5_ (29.06%) and NM_6_ (25.47%). Unlike I-Phellandrene, beta-Phellandrene was not detected in any of the mother plants or control seedlings. However, it was present in all the selected putative mutant seedlings (highest in NM_1,_ 29.53%). Furthermore, Cis-Ocimene, which was another most abundant monoterpene ranging from 16.16% to 21.27% in the mother plants and control samples, was detected only in one of the putative mutant samples, (NM_4_ 17.37%) (**Table 1**). Many of the sesquiterpenes were detected only in the putative mutants such as alpha-Cubebene, trans-Caryophyllene, Germacrene, alpha-Amorphene, Aromadendrene, delta-Cadinene, alpha-Copaene, alpha-Lonipinene, and (−)-ISOLEDENE (**Table 1**). In the present study, in general, the concentration of monoterpenes was more than sesquiterpene in all the samples except putative mutant sample NM_4_, where sesquiterpenes dominated the overall volatile profile (59.14%). Aldehyde (trans-2-Hexenal) was observed to be present only in putative mutant samples NM_1_, NM_4_, and NM_6_ among the studied samples, while alcohol (1-Hexyn-3-ol) was present in the putative mutant sample NM_2_.

**Table 1:** Compositions and mean relative content (area %) of leaf volatile compounds of mother plants (NMP1, NMP2), control samples (NC1, NC2, and NC3), and selected putative mutant samples (NM1, NM2, NM3, NM4, NM5, and NM6) of Nekkare (data are mean of three biological replicates; ND, not detected).

Volatile Compound	Mother plants		Control			Mutants					
	NMP1	NMP2	NC1	NC2	NC3	NM1	NM2	NM3	NM4	NM5	NM6
Alcohols											
1-Hexyn-3-l	ND	ND	ND	ND	ND	ND	1.38	ND	ND	ND	ND
Aldehydes											
trans-2-Hexenal	ND	ND	ND	ND	ND	0.13	ND	ND	0.44	ND	0.34
Mono-terpenoids											
α-Pinene	ND	18.48	13.80	17.31	19.15	32.83	ND	0.09	8.15	8.80	13.23
Camphene	ND	ND	ND	ND	ND	ND	15.83	22.52	ND	ND	ND
Sabinene	1.04	1.06	1.35	1.15	1.83	ND	ND	ND	0.42	0.55	0.89
trans-Ocimene	ND	ND	ND	ND	ND	ND	ND	ND	ND	0.31	0.85
beta-Ocimene	ND	ND	ND	ND	ND	4.19	1.68	2.08	ND	ND	ND
I-Phellandrene	20.45	21.86	23.32	24.95	24.88	ND	1.54	ND	0.34	29.06	25.48
3-Carene	ND	ND	ND	ND	ND	2.25	ND	1.76	ND	ND	ND
beta-Phellandrene	ND	ND	ND	ND	ND	29.53	22.62	29.13	2.34	13.06	22.20
Cis-Ocimene	21.27	16.17	20.44	20.81	18.95	ND	ND	ND	17.37	ND	ND
trans-Ocimene	ND	ND	2.44	ND	ND	21.71	21.56	21.59	6.14	ND	ND
γ-Terpinene	ND	ND	ND	ND	ND	0.65	0.56	ND	ND	ND	ND
α-Terpinolene	ND	ND	ND	ND	ND	0.25	0.19	ND	ND	ND	ND
**Total Monoterpenoids (%)**	**42.76**	**57.57**	**61.35**	**64.22**	**64.80**	**91.41**	**63.98**	**77.17**	**34.76**	**51.78**	**62.64**
Sesqui-terpenoids											
alpha-Gurjunene	18.92	17.93	17.07	16.62	16.03	0.51	6.98	2.07	5.78	0.24	6.73
alpha-Cubebene	ND	ND	ND	ND	ND	0.15	0.32	ND	ND	ND	ND
trans-Caryophyllene	ND	ND	ND	ND	ND	0.10	ND	0.82	30.18	18.28	ND
Germacrene	ND	ND	ND	ND	ND	ND	ND	ND	0.11	0.15	ND
alpha-Amorphene	ND	ND	ND	ND	ND	6.98	ND	ND	ND	ND	ND
Aromadendrene	ND	ND	ND	ND	ND	ND	ND	ND	0.09	0.12	ND
α-Guaiene	ND	ND	ND	ND	ND	0.16	ND	ND	3.86	6.01	0.75
beta-Cadinene	1.42	1.72	1.97	1.68	1.28	ND	1.87	1.14	ND	ND	1.64
delta-Cadinene	ND	ND	ND	ND	ND	0.55	ND	ND	ND	ND	0.59
alpha-Copaene	ND	ND	ND	ND	ND	ND	24.09	17.41	0.26	0.83	23.29
alpha-Humulene	11.89	10.84	9.20	10.37	9.29	ND	ND	ND	16.46	10.50	0.41
beta-Selinene	12.79	ND	10.40	7.11	8.61	ND	ND	1.21	ND	9.08	1.19
Valencene	ND	ND	ND	ND	ND	ND	ND	ND	2.32	2.91	1.97
alpha-Lonipinene	ND	ND	ND	ND	ND	ND	1.24	0.18	ND	ND	0.44
(−)-ISOLEDENE	ND	ND	ND	ND	ND	ND	0.13	ND	0.06	0.10	ND
**Total Sesqui-terpenoids (%)**	**45.01**	**30.48**	**38.65**	**35.78**	**35.20**	**8.46**	**34.64**	**22.83**	**59.14**	**48.22**	**37.01**

### 3.2 Cluster analysis

**Figure 1:**
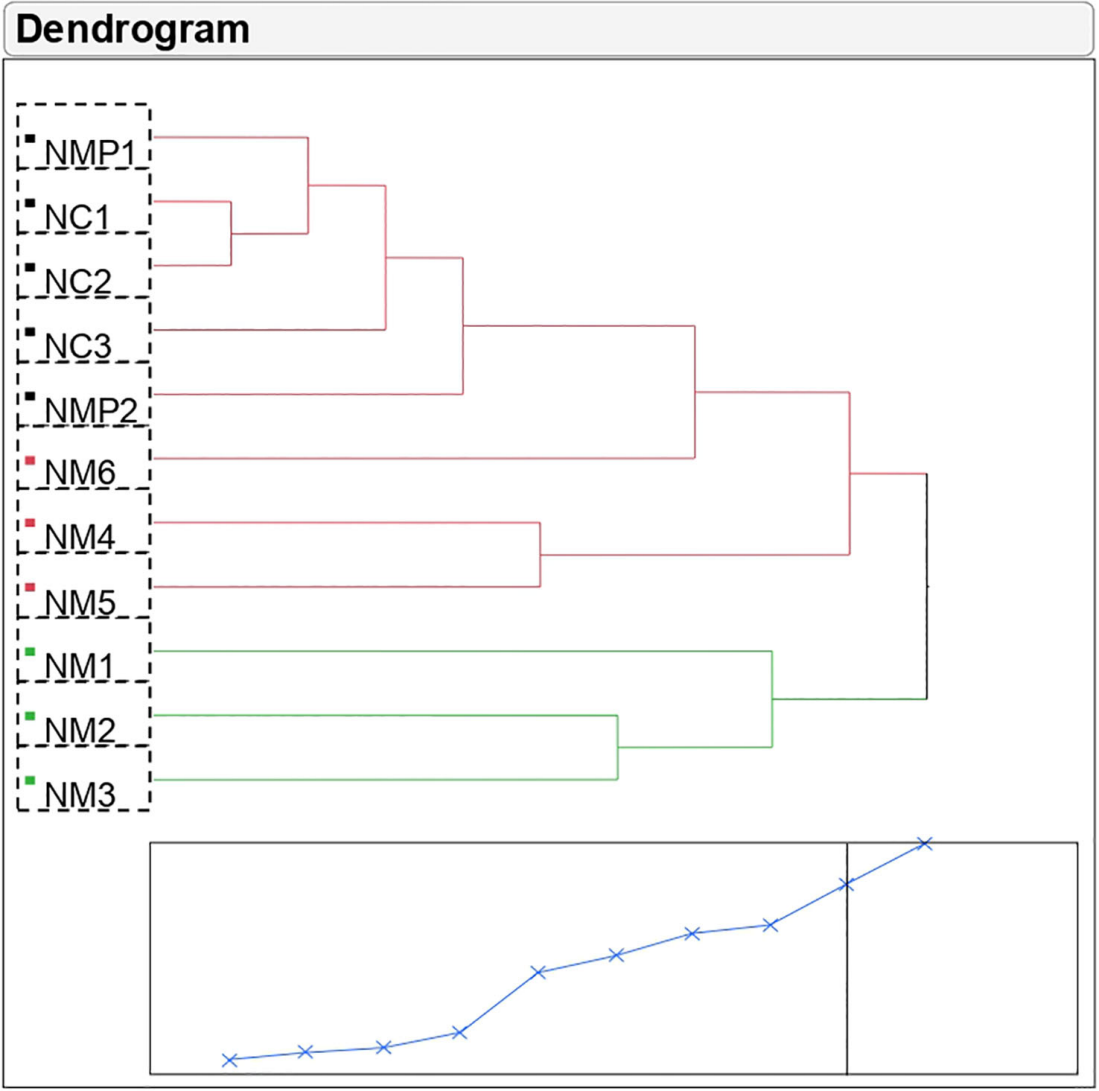
Cluster analysis of Nekkare mother plants (NMP_1_ and NMP_2_), control sample (NC_1_, NC_2_, and NC_3_), and selected putative mutant samples (NM_1_, NM_2_, NM_3_, NM_4_, NM_5_, and NM_6_).

Cluster analysis showed high variability among the selected putative mutant samples; NM_1_, NM_2_, and NM_3_ were different from the mother plants and control seedlings but were similar to each other with respect to composition of volatile compounds (**Figure 1**; **Supplementary Table S2**). The genetic distance was lowest between samples NC_1_ and NC_2_ (0.97) followed by NMP_1_ and NC_1_ (1.18), while among the putative mutant samples, NM_6_ was genetically closest to mother plants and control samples. Using the volatile composition of different samples, a dendogram was generated to understand the relationship between them. The studied samples were divided into two main clusters with two sub-clusters in the first cluster. Cluster 1 comprised of eight samples including mother plants, control, and three putative mutants. Out of these, samples NMP_1_, NC_1_, NC_2_, NC_3_, NMP_2_, and NM_6_ were present in the first sub-cluster, while the second sub-cluster comprised of two putative mutants, *viz.*, NM_4_ and NM_5_. Cluster 2 comprised of putative mutants NM_1_, NM_2_, and NM_3_, suggesting that these three putative mutants are more distant from mother plants and control seedlings as compared to the other three putative mutants (NM_4_, NM_5_, and NM_6_).

### 3.3 Principal component analysis

**Figure 2:**
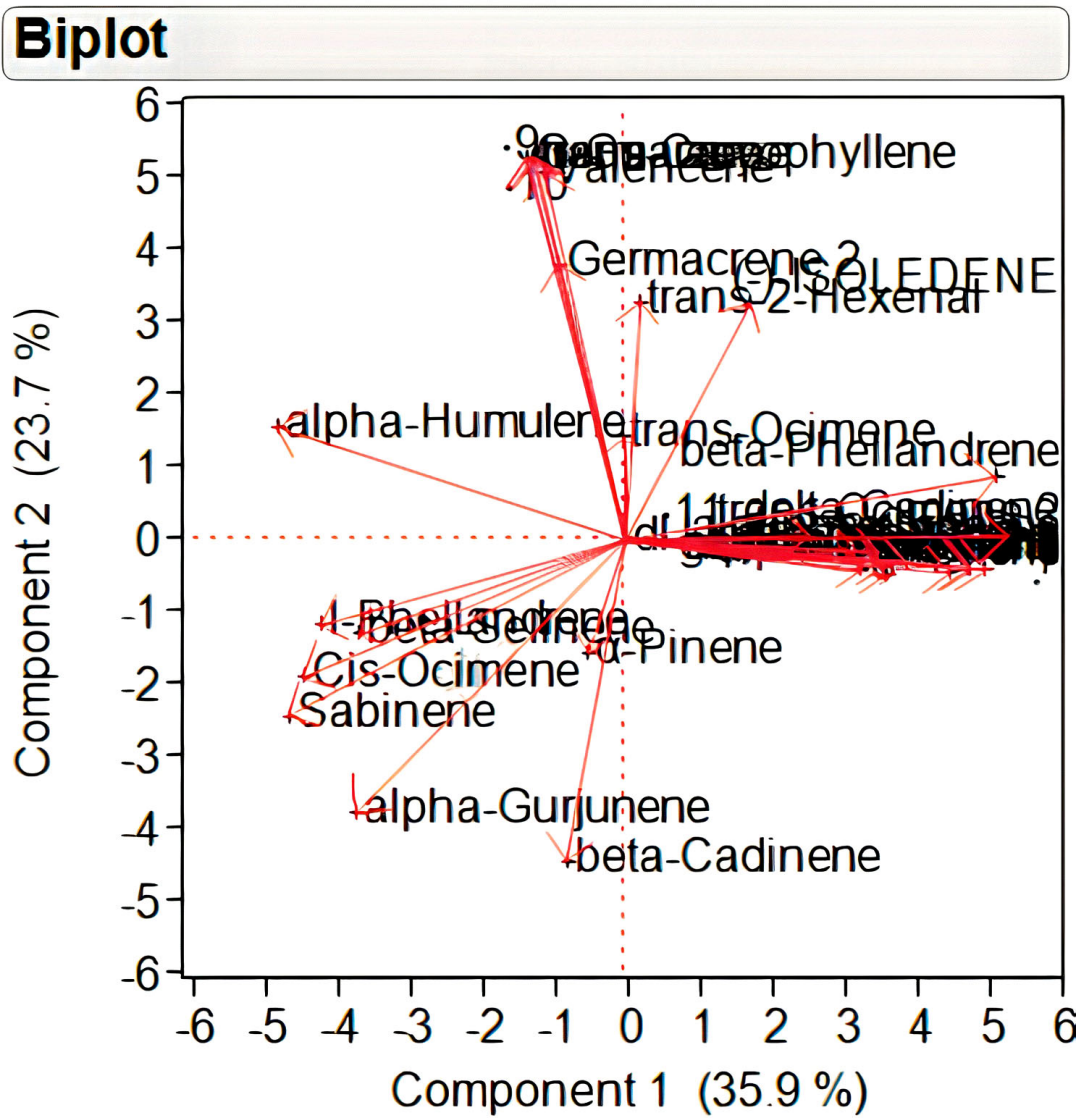
Two-dimensional principal component analysis biplot of individual volatile compounds in Nekkare mother plants (NMP_1_ and NMP_2_), control sample (NC_1_, NC_2_, and NC_3_), and selected putative mutant samples (NM_1_, NM_2_, NM_3_, NM_4_, NM_5_, and NM_6_).

A total of 29 volatile compounds detected in different samples under study were subjected to principal component analysis (PCA) based on a matrix of Pearson correlation coefficients (p<0.05) (**Supplementary Table S3**). PCA results indicated that the total variability is being explained by 29 principal components out of which the components <2.0 eigenvalue were ignored. A principal component loading of more than 0.50 was considered significant for each factor. The correlation matrix, eigenvalues, and factor loadings are provided as **Supplementary Tables S3–S5**. The first four principal components with eigenvalue >2 together explained 85.29% of the total variability in VC. The first two principal components that collectively explained 59.60% of the total variability was depicted as two-dimensional biplot (**Figure 2**). Out of these, 35.9% of total variation was explained by the first principal component, which had an eigenvalue of 10.78. A total of 17 volatile compounds (with loading of >0.5) significantly contributed to the variation in this principal component. Among these, 11 volatile compounds showed positive loadings wherein highest positive loading (>0.8) was exhibited by monoterpenes, *viz.*, beta-Ocimene, gamma terpinene, and alpha-Terpinolene. The second principal component with an eigenvalue of 7.11 explained 23.71% of total variation. Sesquiterpenes like Germacrene, Aromadendrene, trans-Caryophyllene, and alpha-Guaiene with positive loading of >0.9 contributed largely to the variation of this principal component.

### 3.4 Characterisation of putative mutant progenies using SSR markers

#### 3.4.1 Genetic diversity parameters on population level

The level of genetic divergence in the putative mutant population was determined using 12 SSR markers, out of which eight showed polymorphism and were thus used for further analysis. The analysis of molecular variance (AMOVA) showed that most of the variance in the sample was attributable to the variation within individuals being 58% (**Table 2**).

**Table 2:** Summary AMOVA table.

Source	Df	SS	MS	Est. Var.	%
**Among Population**	1	8.741	8.741	0.097	3%
**Among Individuals**	46	193.957	4.216	1.202	39%
**Within Individuals**	48	87.000	1.813	1.813	58%
**Total**	95	289.698		3.111	100%

**Figure 3:**
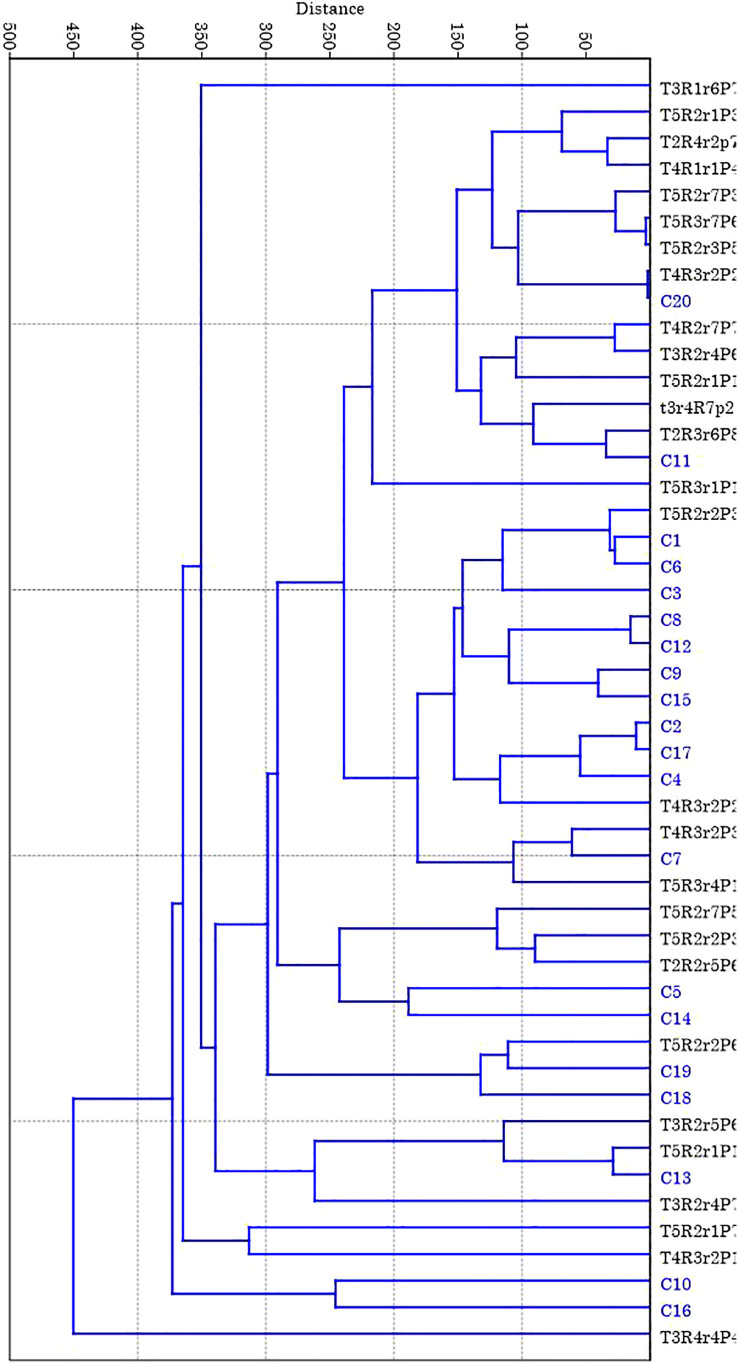
Dendogram of 20 putative mutants (35 Gy gamma irradiation) and 20 control seedlings of polyembryonic mango genotype Nekkare cultivars based on SSR data. The phylogenetic tree obtained from unweighted pair-group method with arithmetic mean (UPGMA) clustering option of the PAST 4.0 software.

Genetic diversity parameters of eight loci for each population are presented in **Table 3**. In the treated population, for different loci, the mean value for number of alleles (Na) was 7 ranging from 4 to 12 (**Table 3**) where the highest number of alleles (12) was recorded for locus MiMRD80. The total number of alleles for the control population was less than that of treated population ranging from 3 for locus MiKVR71 to 12 for locus MiMRD80 with a mean of 6.25. Mean number of effective alleles (Ne) was found to be 3.77 and 3.61 for treated and control populations, respectively. The mean Shannon’s Information Index (I), an important measure of genetic diversity, was maximum (1.50) for the treated population followed by control (1.42). In the treated population, out of eight loci, for five loci, *viz.*, MiIIHR99, MiKVR98, MiMRD88, MiIIHR78, and 21478, Ho was found to be lower than He, while for other three loci, Ho was found to be higher than He. Contrarily, in the control population for all the loci, Ho was lower than He, except locus MiKVR71 where Ho (0.95) was higher than He (0.596). However, irrespective of the samples, Ho was lower than He, and mean Ho was recorded to be 0.48 and 0.46, while mean He was 0.72 and 0.68 for treated and control plants, respectively. In the treated population, negative fixation index was recorded for three loci, and contrastingly, in the control population, negative fixation index was recorded only for one locus, *viz.*, MiKVR71 (−0.593) (**Table 3**). Phylogenetic analysis resulted in the separation of studied samples into two main clusters, *viz.*, I and II (**Figure 3**). Cluster I comprised of only one putative mutant, while cluster II separated into various sub-clusters forming specific groups. A clear separation between clustering of putative mutants and control plants was observed (**Figure 3**), suggesting that mutation could bring about changes in the treated population.

**Table 3:** Number of alleles (Na), number of effective alleles (Ne), Shannon’s Information Index (I), observed heterozygosity (Ho), expected heterozygosity (He), and fixation index (F) for different loci in treated (35Gy gamma irradiation) and control (untreated) seedlings of polyembryonic mango genotype Nekkare (n=20).

Population		8095	MiIIHR78	MiKVR71	MiMRD80	MiKVR99	MiKVR98	MiMRD88	21478	Mean
**Treated**	**Na**	4	7	4	12	7	6	6	10	7.00
	**Ne**	3.941	2.061	2.834	3.547	4.614	4.364	4.226	4.579	3.77
	**I**	1.379	1.105	1.153	1.667	1.684	1.583	1.577	1.861	1.50
	**Ho**	0.815	0.077	0.962	0.852	0.5	0.083	0.5	0.08	0.48
	**He**	0.746	0.515	0.647	0.718	0.783	0.771	0.763	0.782	0.72
	**F**	-0.092	0.851	-0.486	-0.186	0.362	0.892	0.345	0.898	0.32
**Control**	**Na**	4	6	3	12	5	6	6	8	6.25
	**Ne**	2.711	1.77	2.477	5.674	3.88	4.73	4.762	2.893	3.61
	**I**	1.101	0.934	0.988	2.079	1.452	1.652	1.656	1.489	1.42
	**Ho**	0.278	0.158	0.95	0.6	0.611	0.167	0.6	0.333	0.46
	**He**	0.631	0.435	0.596	0.824	0.742	0.789	0.79	0.654	0.68
	**F**	0.56	0.637	−0.593	0.272	0.177	0.789	0.241	0.491	0.32

## 4 Discussion

### 4.1 Volatile profiling

Volatile organic compounds including aldehydes, ketones, esters, and terpenes conferring aroma are among the major determinant of fruit quality. Terpene hydrocarbons including terpinolene, 3-carene, caryophyllene, and α-Pinene are reported to be the major volatiles (depending upon the cultivars) in the pulp of mango fruit (Li et al., 2017). The analysis of volatile compounds in the leaves of polyembryonic mango genotype Nekkare showed the differences between gamma irradiation-derived and untreated plants. Mango volatile profile has been reported to be contributed by volatile compounds like monoterpenes, sesquiterpenes, alcohols, lactones, aldehydes, esters, ketones, fatty acids, and carotenoid compounds (Pandit et al., 2009; Li et al., 2017). In this study, monoterpenes and sesquiterpenes were found to be the main volatile compounds present in the studied samples. However, significant differences were observed in the compositions and proportions of terpenes in the studied samples. On the basis of abundance of different terpenes, Andrade et al. (2000) classified mango genotypes into terpinolene, 3-carene, and myrcene genotypes. In the selected putative mutant samples, the number of monoterpenes was more ranging from 5 (in NM_5_ and NM_6_) to 7 (in NM_1_ and NM_2_) as compared to mother plants and control seedlings where it ranged from 3 (in NMP_1_) to 5 (in NC_1_). Earlier studies have also confirmed terpenes as the most abundant volatile constituent of mango (Pino et al., 2005; Li et al., 2017). The number of sesquiterpenes detected in the selected putative mutants was more ranging from 6 (in NM_1_, NM_2_ and NM_3_) to 10 (NM_5_) in comparison to mother plants and control samples where it ranged from 3 (NMP_2_) to 4 (NMP_1_, NC_1_, NC_2_, and NC_3_). Among the sesquiterpenes, only alpha-Gurjunene was found to be present in all the samples under study including mother plants, control samples, and selected putative mutants. However, the amount detected in mother plants and control samples was more than in the putative mutants. In a study, all the sesquiterpene-dominated mango cultivars were found to be of Indian origin (Pandit et al., 2009). Oliveira et al. (2010) reported differences in volatile composition of white and dark varieties of fig and found sesquiterpenes, Germacrene D, beta-caryophyllene, and s-elemene to be the main volatile compounds in the leaves of the studied fig cultivars. Furthermore, in the present study, aldehyde (trans-2-Hexenal) was found to be present only in putative mutant samples NM_1_, NM_4_, and NM_6_ among all the studied samples. Although present in lower concentrations, aldehydes and alcohols contribute significantly towards flavour and aroma to mangoes (Pino et al., 2005). A very little concentration of aldehyde was detected in two of 25 cultivars studied being 0.38% in Chinese cultivar Renong No.1 and 0.01% in Indonesian cultivar Gleck (Li et al. 2017). Aldehydes break down to alcohols and lactones, which further contribute to fruit aroma. In this study, no lactones were detected, but alcohol (1-Hexyn-3-ol) was present in the putative mutant sample NM_2_.

### 4.1 Characterisation of putative mutant progenies using SSR markers

High level of morphological and stomatal diversity was observed in the irradiated population of polyembryonic mango genotype Nekkare (Perveen et al., 2022). Genetic divergence in the putative mutant population was estimated by using 12 SSR markers. Out of the 12 markers, eight showed polymorphism and were thus used for further analysis. The mean total number of alleles for the treated population was found to be more than that of control population. The high allelic richness observed suggests that mutation treatment could bring about variability in the treated population. An increase in the mean number of alleles with increase in dosage of irradiation has previously been reported (Taheri et al., 2016). The mean Shannon’s Information Index (I), an important measure of genetic diversity, was also recorded and was highest for treated population as compared to control. The higher value of I reflects the effectiveness of SSR markers used to identify the variation created by irradiation treatment. Several reports of utilisation of molecular markers for the detection of genetic diversity in mutant population are available in different fruit crops like citrus (Sülü et al., 2020), grapes (Dev et al., 2021), olive (Tekin et al., 2022), and pomegranate (Perveen et al., 2023b). SSR markers have been widely preferred for germplasm characterisation and phylogenetic studies in mango (Azam et al., 2018; Azam et al., 2019; Arogundade et al., 2022). Hitherto, reports on molecular characterisation of mutant population of mango are very few, and results of this study showed the effectiveness of SSR markers for determining the variability in putative mutant population of Nekkare. However, further research using more number of polymorphic SSR markers is paramount to validate the findings of the present study before using these results in devising mango rootstock breeding programme. The selected putative mutants are being maintained and will be evaluated in ensuing studies.

## 5 Conclusion

The studied samples of polyembryonic mango genotype Nekkare, including mother plants, control, and selected putative mutants differed in total concentration and compositions of major volatile compounds, allowing us to distinguish between gamma radiation-treated and untreated plants. Monoterpenes and sesquiterpene hydrocarbons were found to be the major volatile compounds of these samples, wherein untreated samples (control and mother plants) were found to be rich in monoterpenes, while an abundance of sesquiterpenes was detected in the putative mutants. Mother plants and control seedlings grouped together, suggesting the similarity between them while the genetically distant putative mutants grouped apart from the mother plants and control seedlings; this could be the result of mutation. High allelic richness and mean Shannon’s Information Index observed in the putative mutant population suggest that mutation created variability in the treated population. Hence, volatile profiling and molecular characterisation using SSR markers could be an effective tool to detect variation in mutated population, and the former can be used to validate putative mutants in polyembryonic mango genotypes where the seedlings are similar to mother plants due to their origin from nucellar region.

## Data availability statement

The original contributions presented in the study are included in the article/**Supplementary Material**. Further inquiries can be directed to the corresponding authors.

## Author contributions

NP, MD, MS, KS, and KR designed the research. NP performed the experimental work under the supervision of KS and KR. RV and HM provided the required software and conducted the statistical analysis. NP wrote the manuscript. NP, MD and HM reviewed and edited the manuscript. All authors contributed to the article and approved the submitted version.
