# Supplementary material for: Volatile profiling as a potential biochemical marker for validation of gamma irradiation derived putative mutants in polyembryonic genotypes of mango (Mangifera indica L.)
Source: Front Plant Sci. 2023 Sep 1;14:1168947. doi: 10.3389/fpls.2023.1168947 (PMC10503045; doi:10.3389/fpls.2023.1168947)
Supplement: Supplementary file 1 [file Table_1.docx]

**Supplementary Table S1.1. Sequences of 8 SSR primer used for the study**

| **SI.No.** | **Locus** | **Primer** | **Expected allele size (bp)** |
| --- | --- | --- | --- |
| **1** | **8095** | **F:** 5’GTAAAACGACGGCCAGTTGAATGGGTAATGGTGAATCG3'  **R:** 5'GTTTCTTTCAACTCAATCTGATCGCTTACA3’ | **167-168** |
| **2** | **MiIIHR99** | **F:** 5'GTAAAACGACGGCCAGTCTTCATCGAATCCAAGGCAT3'  **R:** 5'GTTTCTTCTTCCATGGCACGAGTAGGT3' | **150-170** |
| **3** | **MiMRD80** | **F:** 5'GTAACGACGGCCAGTTTAAAGGGGCTGACAAATGG3'  **R:** 5'GTTTCTTGTGGAGCACTGATTTTTGGG3' | **153-155** |
| **4** | **MiKVR71** | **F:** 5'TAAAACGACGGCCAGTAAAAGTGCCACAGAAAACATGTAA3'  **R:** 5'GTTTCTTATGCCTCAACCTGTTATGCC3' | **166-168** |
| **5** | **MiKVR98** | **F:** 5'GTAAAACGACGGCCAGTTGATTTCCCCTTAATGTTGATG3'  **R:** 5'GTTTCTTTCATTATCATGAGTTTCGCCA3' | **140-170** |
| **6** | **21478** | **F:** 5'GTAAAACGACGGCCAGTAAACTTTAATGGATTTTATCGTGTAAT3'  **R:** 5'GTTTCTTAAATACCCCACCCATTTTGC3' | **130-160** |
| **7** | **MiIIHR78** | **F:** 5'GTAAAACGACGGCCAGTGCCTTGAGGAGGTTTTGACA3'  **R:** 5'GTTTCTTGATGCTTCTTTCCCAACAGC3' | **167-168** |
| **8** | **MiMRD88** | **F:** 5'GTAAAACGACGGCCAGTAAAATGGACGCCACAAAGTG3'  **R:** 5'GTTTCTTGTTTCGGATTTCTCATGGGA3' | **130-150** |
