# Supplementary material for: Volatile profiling as a potential biochemical marker for validation of gamma irradiation derived putative mutants in polyembryonic genotypes of mango (Mangifera indica L.)
Source: Front Plant Sci. 2023 Sep 1;14:1168947. doi: 10.3389/fpls.2023.1168947 (PMC10503045; doi:10.3389/fpls.2023.1168947)
Supplement: Supplementary file 2 [file Table_2.docx]

**Supplementary Table S1.2. PCR reactions for molecular markers used in the present study**

| **Components** | **Concentration** | **PCR reaction (20 µl)** |
| --- | --- | --- |
| DNA | 50ng/ µl | 4 µl |
| PCR water |  | 10.2µl |
| Buffer | 10X | 2 µl |
| dNTP | 1mM | 1 µl |
| Forward Primer | 10 pM | 0.5µl |
| Reverse Primer | 10 pM | 1µl |
| Labelled M13 Probe (FAM, VIC, NED and PET) | 5 pM | 0.5µl |
| Taq Polymerase |  | 0.8µl |
| **Total** |  | **20.0 µl** |
