# Supplementary material for: Volatile profiling as a potential biochemical marker for validation of gamma irradiation derived putative mutants in polyembryonic genotypes of mango (Mangifera indica L.)
Source: Front Plant Sci. 2023 Sep 1;14:1168947. doi: 10.3389/fpls.2023.1168947 (PMC10503045; doi:10.3389/fpls.2023.1168947)
Supplement: Supplementary file 3 [file Table_3.docx]

**Supplementary Table S1.3. PCR protocol followed for microsatellite primers**

| **Sl. No.** | **Steps** | **Microsatellite Primers** | | **Cycles** |
| --- | --- | --- | --- | --- |
|  |  | **Temperature (°C)** | **Time** |  |
| 1 | Initial denaturation | 94 | 3 min | 35 cycles |
| 2 | Denaturation | 94 | 30 sec |  |
| 3 | Annealing | 55-60***** | 30 sec |  |
| 4 | Primer extension | 72 | 30 sec |  |
| 5 | Final extension | 72 | 5 min |  |
| 6 | Hold | 4 | ∞ |  |

***Specific annealing temperature as per the Primers**
